# Multilocus sequence typing reveals diverse known and novel genotypes of *Leptospira* spp. circulating in Sri Lanka

**DOI:** 10.1371/journal.pntd.0008573

**Published:** 2020-08-25

**Authors:** Lilani Karunanayake, Chandika D. Gamage, Chandima P. Gunasekara, Sajiv De Silva, Hidemasa Izumiya, Masatomo Morita, Devinda S. Muthusinghe, Kumiko Yoshimatsu, Roshan Niloofa, Panduka Karunanayake, Wimalasiri Uluwattage, Makoto Ohnishi, Nobuo Koizumi

**Affiliations:** 1 National Reference Laboratory for Leptospirosis, Department of Bacteriology, Medical Research Institute, Colombo, Sri Lanka; 2 Department of Microbiology, Faculty of Medicine, University of Peradeniya, Kandy, Sri Lanka; 3 Base Hospital, Elpitiya, Sri Lanka; 4 Department of Bacteriology I, National Institute of Infectious Diseases, Shinjuku, Tokyo, Japan; 5 Graduate School of Infectious Diseases, Hokkaido University, Sapporo, Hokkaido, Japan; 6 Institute of Genetic Medicine, Hokkaido University, Sapporo, Hokkaido, Japan; 7 Institute of Biochemistry, Molecular Biology and Biotechnology, University of Colombo, Sri Lanka; 8 Department of Clinical Medicine, University of Colombo, Sri Lanka; 9 Teaching Hospital, Karapitiya, Sri Lanka; University of Connecticut Health Center, UNITED STATES

## Abstract

**Background:**

Leptospirosis has gained much attention in Sri Lanka since its large outbreak in 2008. However, most of the cases were clinically diagnosed and information on *Leptospira* genotypes and serotypes currently prevailing in the country is lacking.

**Methodology/Principal findings:**

We retrospectively analyzed 24 *Leptospira* strains from human patients as well as isolated and characterized three *Leptospira* strains from black rats using the microscopic agglutination test with antisera for 19 serovars and multilocus sequence typing. The isolates were identified as *Leptospira borgpetersenii* sequence types (STs) 143 and 144; *L*. *interrogans* STs 30, 34, 43, 44, 74, 75, 80, 308, 313, 314, 316, and 317; and *L*. *kirschneri* ST318. Six of the 15 STs were identified for the first time in this study. Five serogroups such as Autumnalis, Grippotyphosa, Hebdomadis, Javanica, and Pyrogenes were detected among the isolates. Contrary to previous studies, various genotypes including novel STs were isolated during an outbreak in Southern Province. *L*. *borgpetersenii* serogroup Javanica ST143 was isolated both from a human and black rat.

**Conclusions/Significance:**

This study revealed that genetically diverse *Leptospira* strains currently circulate in Sri Lanka: some genotypes have been circulating and others have emerged recently, which may explain the recent surge of leptospirosis patients with varying clinical manifestations and frequent outbreaks of leptospirosis. Black rats were identified as the source of infection for humans, but reservoir animals for other genotypes remain unknown.

## Author summary

Leptospirosis is an important but largely under-recognized public health problem in the tropics. It is a re-emerging disease and outbreaks of leptospirosis have been frequently reported in Sri Lanka. Most of the cases, however, were clinically diagnosed, and *Leptospira* serotypes and genotypes currently prevailing in the country remain unknown. For the prevention of leptospirosis, doxycycline prophylaxis and health education (early medical help, use of protective wear, etc) are implemented in farmers, which is the highest risk group, in endemic areas. In the present study, we revealed that genetically diverse *Leptospira* strains presently cause human leptospirosis in this country. Novel STs were isolated during an outbreak in Southern Province and may be related to the recent surge in leptospirosis patients with new clinical entities. The emergence of new STs also implies the importance of strict quarantine for imported domestic animals that are potential reservoirs of *Leptospira* spp. as well as the unintentional introduction of rodent reservoirs and the existence of unidentified reservoir animals in this country. New information from this study will be valuable in future research for patient management and specific targeted control approaches for reservoir hosts in the prevention and control of leptospirosis in Sri Lanka.

## Introduction

Leptospirosis is a zoonotic disease caused by pathogenic spirochaetes of *Leptospira* spp. belonging to subclades P1 and P2 [1–4]. Human leptospirosis is an acute febrile illness with an extremely broad clinical spectrum ranging from mild influenza-like illness to severe disease forms characterized by jaundice, bleeding, renal failure, and death [1, 3]. Approximately one million cases and 58,900 deaths of leptospirosis are estimated to occur worldwide each year, more than 70% of which are reported from the tropical, poorest regions of the world [5]. Pathogenic *Leptospira* spp. colonize the proximal renal tubules and are excreted in the urine of reservoir animals [1, 3]. Human leptospirosis is primarily transmitted by exposure to water or soil contaminated with the urine of infected animals [1, 3].

Leptospirosis is a re-emerging disease in Sri Lanka and has gained much attention since the large outbreak in 2008 [6]. A recent systematic review indicated that the cumulative annual incidence of leptospirosis that required hospitalization from 2008 to 2015 was 52.1 per 100,000 people, with an estimated case fatality ratio of 7.0% [7]. A wide array of new clinical entities such as pulmonary hemorrhage, pancreatitis, and myocarditis with high case fatality were seen in recent outbreaks [8–11]. Most of the cases, however, were clinically diagnosed, and some were diagnosed serologically by the microscopic agglutination test (MAT) using a non-pathogenic *Leptospira* strain Patoc I [12]. Recent studies performed MAT using a panel of *Leptospira* serovars and detected antibodies to various leptospiral serovars in human patients, rodents, and cattle [11, 13–15]. In addition, recent molecular investigations detected *L*. *borgpetersenii*, *L*. *interrogans*, *L*. *kirschneri*, and *L*. *weilii* in clinical samples of febrile human patients and regional differences of predominant *Leptospira* species in the country [13, 15–18]. *Leptospira* DNAs belonging to *L*. *borgpetersenii*, *L*. *interrogans*, and *L*. *kirschneri* have been recently detected in cattle kidney or urine samples collected in distinct areas [14, 19]. Although *Leptospira* strains were isolated from humans, rodents, shrews, and dogs in 1960s and early 1970s [20, 21], only one report of culture isolation of *L*. *interrogans* unidentified serogroups has been published in Sri Lanka during the past 40 years [22]. Therefore, *Leptospira* genotypes and serotypes currently prevailing in the country remain largely unknown.

In the present study, we characterized *Leptospira* isolates from clinically-suspected leptospirosis patients and rats in various Provinces in Sri Lanka using the microscopic aggulutination test with antisera for 19 serovars and multilocus sequence typing (MLST).

## Methods

### Maintenance of *Leptospira* isolates from clinically-suspected leptospirosis patients

We retrospectively analyzed 24 *Leptospira* strains stored at National Reference Laboratory for Leptospirosis, Medical Research Institute, Sri Lanka. These strains were isolated from clinically-suspected leptospirosis patients defined by the National Guideline on Management of Leptospirosis 2016, Ministry of Health, Sri Lanka (www.epid.gov.lk) between 2012 to January 2019 by bedside inoculation of two drops of fresh blood into liquid Ellinghausen–McCullough–Johnson–Harris (EMJH) medium that comprised Difco Leptospira Medium Base EMJH and Difco Leptospira Enrichment EMJH (Becton, Dickinson and Company, Franklin Lakes, NJ) with no antibiotics. During the period, 401 blood cultures were collected and 28 *Leptospira* strains were isolated (isolation rate: 7.0%), of which four cultures were contaminated and not included in this study. The isolates were stored in semisolid EMJH as described above at ambient temperature and inoculated into liquid medium before serological and molecular characterization and cultured at 30°C. This study was approved by the Ethics Review Committee of Medical Research Institute.

### Isolation of *Leptospira* spp. from small mammals

For the survey of reservoir animals, we used live traps and captured 108 rodents and shrews in Kandy, Central Province in 2015 and 116 rodents in Giradurukotte, Uva Province in 2017. Animal dissections were performed according to the American Veterinary Medical Association guidelines under the ethical approval of the Institutional Review Board of the University of Peradeniya. The species of animals captured was identified by DNA sequencing of the mitochondrial cytochrome b gene, *cytb* [23]. DNA was extracted from animal kidney tissues using DNAZOL Reagent (Invitrogen, Carlsbad, CA). *cytb* was amplified with AmpliTaq Gold 360 Mater Mix (Applied Biosystems, Foster City, CA) using primer pairs H15300 (5′-GTTTACAAGACCAGAGTAAT-3′) and L497A (5′-CCTAGTAGAATGAATCTGAGG-3′) and H655A (5′-TGTGTAGTATGGGTGGAATGG-3′) and L14115 (5′-GACATGAAAAATCATCGTTG-3′) [23] or L14274 (5′-CGAAGCTTGATATGAAAAACCATCGTTG-3′)and H15149 (5′-AAACTGCAGCCCCTCAGAATGATATTTGTCCTCA-3′) [24]. After an initial denaturation step at 95°C for 10 min, the reaction mixture was subjected to 35 cycles of denaturation at 95°C for 30 s, annealing at 50°C for 30 s, and extension at 72°C for 60 s, followed by a final extension at 72°C for 5 min. DNA sequencing of the amplicons was performed using the BigDye Terminator v3.1 Cycle Sequencing Kit (Applied Biosystems, Foster City, CA) with the PCR primers and the resulting sequences were subjected to phylogenetic analyses as previously described [23]. All black rats captured in this study belonged to *Rattus rattus* complex lineage 1 [25]. For the isolation of *Leptospira* spp., kidney tissues were inoculated into 5 ml liquid EMJH medium that composed Difco Leptospira Medium Base EMJH and Difco Leptospira Enrichment EMJH (Becton, Dickinson and Company, Franklin Lakes, NJ) and supplemented with 100 μg/mL of 5-fluorouracil and cultured at 30°C for 3 months.

### Serogroup identification of *Leptospira* isolates

Serogroups of the isolates were identified by the MAT using a panel of anti-*Leptospira* rabbit sera for serogroups Australis (serovar Australis), Autumnalis (serovars Autumnalis and Rachmati), Ballum (serovar Castellonis), Bataviae (serovar Bataviae), Canicola (serovar Canicola), Cynopteri (serovar Cynopteri), Djasiman (serovar Djasiman), Grippotyphosa (serovar Grippotyphosa), Hebdomadis (serovar Hebdomadis), Icterohaemorrhagiae (serovar Icterohaemorrhagiae), Javanica (serovar Javanica), Mini (serovar Mini), Pomona (serovar Pomona), Pyrogenes (serovar Pyrogenes), Sarmin (serovar Sarmin), Sejroe (serovars Hardjo and Sejroe), and Tarassovi (serovar Tarassovi). Twenty-five microliters of 4- to 7-day-cultures of the isolates were incubated with the same volume of 200-fold diluted antisera for 2.5 hr at 30°C and the reaction was interpreted as positive when the proportion of free, unagglutinated leptospires was < 50% compared with the control suspension. When no positive reactions were observed, MAT was conducted using 50-fold diluted antisera. Each isolate except for serogroup Autumnalis strains (MRI-G12-13, P11-15, U9-17, and X5-18) reacted with a single antiserum and no cross reactions were observed. The serogroup Autumnalis strains reacted with anti-Autumnalis and Rachmati antisera.

### Molecular characterization of *Leptospira* isolates

DNA was extracted from 0.5 ml cultures of the isolates using the DNeasy Blood and Tissue Kit (Qiagen, Hilden, Germany). Partial *flaB* sequence was amplified with *TaKaRa Ex Taq* Hot Start Version (Takara Bio, Japan) using primers L-*flaB*-F1 (5′-CTCACCGTTCTCTAAAGTTCAAC-3′) and L-*flaB*-R1 (5′-TGAATTCGGTTTCATATTTGCC-3′) for species classification [26]. After an initial denaturation step at 95°C for 1 min, the reaction mixture was subjected to 30 cycles of denaturation at 98°C for 10 s, annealing at 50°C for 30 s, and extension at 72°C for 50 s. DNA sequencing of the amplicons was performed using the BigDye Terminator v3.1 Cycle Sequencing Kit (Applied Biosystems, Foster City, CA, USA) with the PCR primers, and the resulting sequences were compared with those of reference strains through BLAST search (https://blast.ncbi.nlm.nih.gov/Blast.cgi). The *flaB* sequences determined have been deposited in a public database (DDBJ accession numbers LC565816– LC565842). MLST was performed using seven housekeeping genes, *glmU*, *pntA*, *sucA*, *tpiA*, *pfkB*, *mreA*, and *caiB*, as previously described by Boonsilp *et al*. [27]. Sequence types (STs) were assigned through the MLST database (https://pubmlst.org/leptospira/). New STs obtained in this study have been deposited in the database (ST308, ST313, ST314, ST316, ST317, and ST318). A minimum spanning tree (MST) based on the allelic profiles of MLST of the isolates was created using BioNumerics Software (version 7.6, Applied-Maths, Sint Maartens-Latem, Belgium) with categorical efficiency.

## Results

### Information on culture-positive population

**Fig 1:**
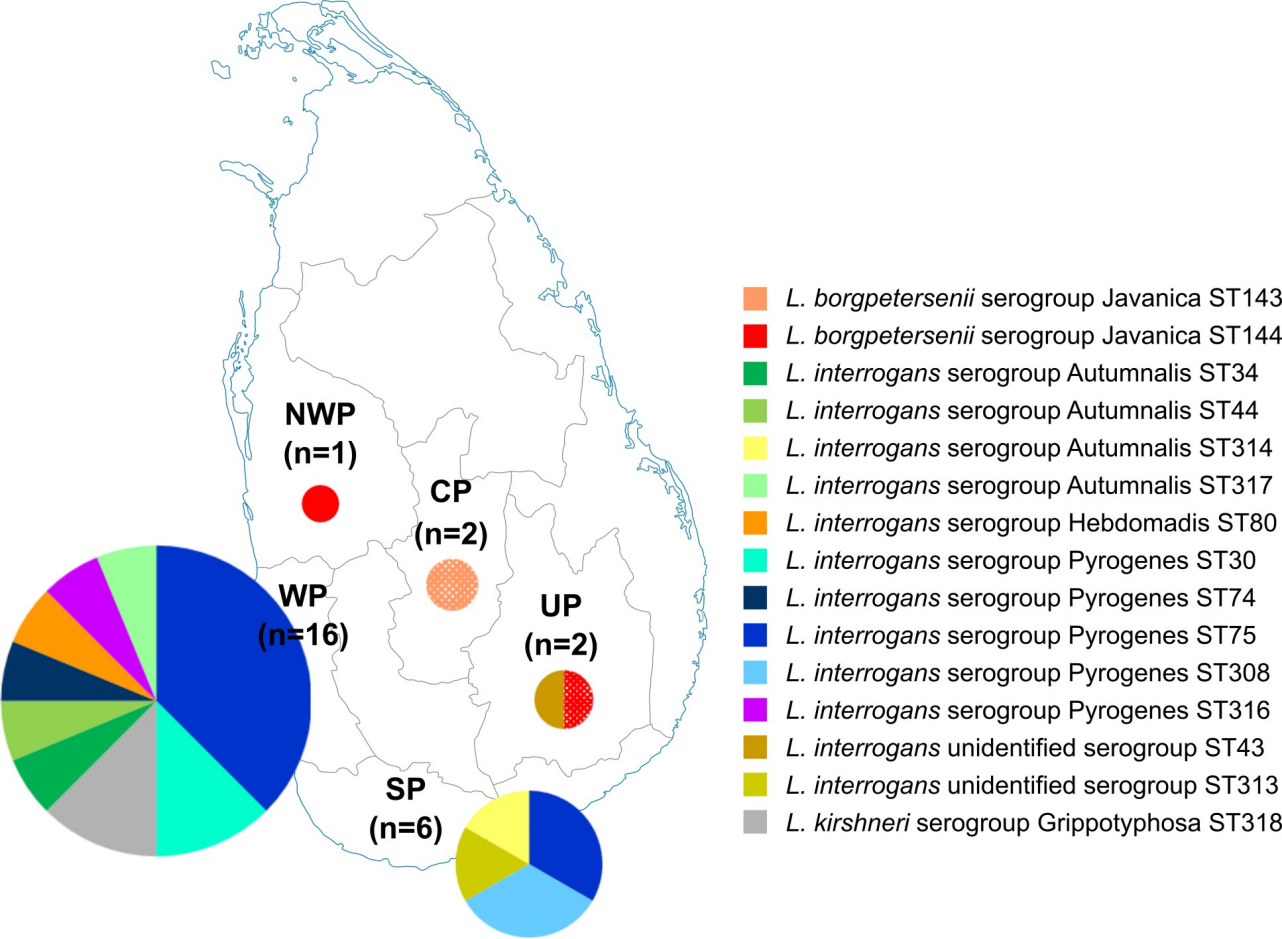
Distribution of 27 *Leptospira* strains isolated in Sri Lanka. One isolate of ST144 in UP and two isolates of ST143 in CP were isolated from rats (indicated by dots in the pie charts), and others were isolated from human patients. CP, Central Province; NWP, North-Western Province; SP, Southern Province; UP, Uva Province; WP, Western Province.

A total of 24 *Leptospira* strains isolated from 401 clinically-suspected leptospirosis patients (positive rate including four contaminated cultures: isolation rate, 7.0%) were analyzed. The median (range) age of the culture-positive population was 39.5 (12–72) years, with 95.8% male predominance (n = 23). Majority (67%, n = 16) were from Western Province, and others were from Southern Province (n = 6), North-Western Province (n = 1) and Uva Province (n = 1) (Fig 1). Among 21 cases with clinical information available (Western Province; 14, Southern Province; 6, North-Western Province; 1), severe symptoms were seen in eight cases, of which six were from the Elpitiya Medical Officer of Health region in Southern Province (S1 Table). All patients with severe disease survived.

### Molecular and serological characterization of *Leptospira* isolates from human patients

By partial *flaB* gene sequencing and MAT using a panel of 19 antisera, species and serogoup of the isolates were identified as *Leptospira interrogans* serogroup Pyrogenes (14 strains), *L*. *interrogans* serogroup Autumnalis (4 strains), *L*. *kirschneri* serogroup Grippotyphosa (2 strains), *L*. *borgpetersenii* serogroup Javanica (1 strain), and *L*. *interrogans* serogroup Hebdomadis (1 strain) (Table 1). Serogroups of two *L*. *interrogans* isolates could not be identified: one of the isolates reacted weakly with anti-serovar Rachmati antiserum at 1:100, and the other reacted with none of the 19 antisera.

**Table 1:** Serological and molecular characterization of *Leptospira* spp. isolated from human patients and rats in Sri Lanka.

Source	Species	Serogroup	ST	Province* (No. of isolates)	Isolate
Human	*Leptospira borgpetersenii*	Javanica	144	NWP (1)	MRI-F11-13
	*L*. *interrogans*	Autumnalis	34	WP (1)	MRI-X5-18
	*L*. *interrogans*	Autumnalis	44	WP (1)	MRI-P11-15
	*L*. *interrogans*	Autumnalis	314^§^	SP (1)	MRI-U9-17
	*L*. *interrogans*	Autumnalis	317^§^	WP (1)	MRI-G12-13
	*L*. *interrogans*	Hebdomadis	80	WP (1)	MRI-K6-14
	*L*. *interrogans*	Pyrogenes	75	SP (2) WP (6)	MRI-B9-12 MRI-E11-13 MRI-H12-14 MRI-J3-14 MRI-N9-14 MRI-S4-17 MRI-T7-17 MRI-A1-1-19
	*L*. *interrogans*	Pyrogenes	30	WP (2)	MRI-A9-12 MRI-L5-14
	*L*. *interrogans*	Pyrogenes	308^§^	SP (2)	MRI-Q11-16 MRI-V9-17
	*L*. *interrogans*	Pyrogenes	74	WP (1)	MRI-D9-13
	*L*. *interrogans*	Pyrogenes	316^§^	WP (1)	MRI-C8-13
	*L*. *interrogans*	Unidentified^†^	43	UP (1)	MRI-O1-15
	*L*. *interrogans*	Unidentified^‡^	313^§^	SP (1)	MRI-R11-16
	*L*. *kirschneri*	Grippotyphosa	318^§^	WP (2)	MRI-I2-14 MRI-W4-18
Rat (*R*. *rattus*)	*L*. *borgpetersenii*	Javanica	143	CP (2)	Rat13 Rat35
	*L*. *borgpetersenii*	Javanica	144	UP (1)	GR92

*CP, Central Province; NWP, North-Western Province; SP, Southern Province; UP, Uva Province; WP, Western Province.

^†^The strain exhibited weak reaction with 100-fold diluted anti-Rachmati antiserum.

^‡^The strain did not react with any of the 19 antisera described in the text.

^§^STs newly assigned in this study.

MLST using seven housekeeping genes revealed 14 STs among human isolates: ST144 (1 strain) in *L*. *borgpetersenii* serogroup Javanica; ST318 (2) in *L*. *kirschneri* serogroup Grippotyphosa; ST34 (1), ST44 (1), ST314 (1), and ST317 (1) in *L*. *interrogans* serogroup Autumnalis; ST80 (1) in *L*. *interrogans* serogroup Hebdomadis; ST30 (2), ST74 (1), ST75 (8), ST308 (2), and ST316 (1) in *L*. *interrogans* serogroup Pyrogenes; and ST43 (1) and ST313 (1) in *L*. *interrogans* unidentified segrogroup strains (Table 1). Among the 14 STs, six STs, ST308, ST313, ST314, ST316, ST317, and ST318 were newly assigned in this study. Three new genotypes, ST308, ST313, and ST314, were isolated from severe patients (S1 Table) during the outbreak from 2015 to 2017 in Elpitiya, Southern Province [9].

**Fig 2:**
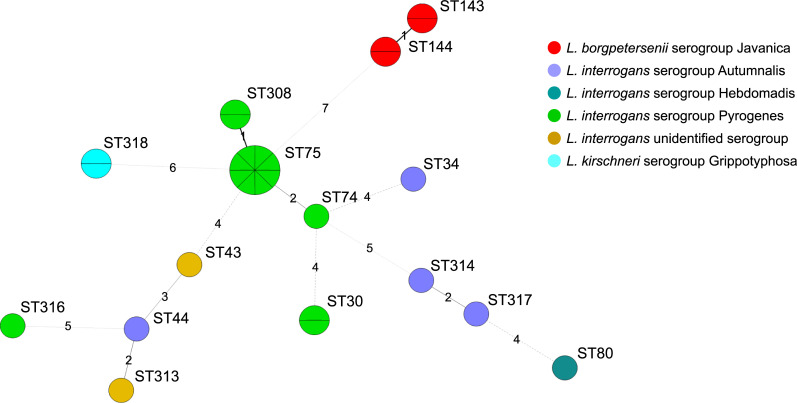
MST of 27 *Leptospira* strains isolated in Sri Lanka based on the allelic profiles of MLST. Each circle represents an individual ST, and the color indicates the *Leptospira* serogroup. Circle size corresponds to the number of isolates in each ST. The digits on the lines between two circles represent the number of different alleles between two STs. One isolate of ST144 and two isolates of ST143 were isolated from rats, and others were isolated from human patients. ST308, ST313, ST314, ST316, ST317, and ST318 were newly assigned in this study.

MST indicated genetic diversity in the same serogroups (Fig 2 and S1–S3 Figs): for example, in *L*. *interrogans* serogroup Pyrogenes, ST74, ST75, and ST308 are of the same group (>5/7 alleles were identical), whereas ST30 and ST316 showed more than four allele differences (Fig 2). Regarding novel STs identified in this study, ST318 is the same group with ST68 (5/7 loci) that was isolated from humans in Thailand (S3 Fig). ST313 is the same group with ST44 (5/7 loci) found in Sri Lanka, but their serogroups are different. On the other hand, there are no known STs that are the same group with STs 314, 316, and ST317, although ST314 and ST317 form the same group (Fig 2 and S2 Fig).

### Survey of reservoir animals

We captured 108 rodents and shrews (*Rattus rattus*, 102; *Suncus murinus*, 6) in Kandy, Central Province and 116 rodents (*R*. *rattus*, 112; *Mus booduga*, 2; *Bandicota bengalensis*, 1; unidentified Murinae species, 1) in Girandurukotte, Uva Province. Three *L*. *borgpetersenii* serogroup Javanica, two from Kandy (1.9%) and one from Girandurukotte (0.9%), were isolated by kidney tissue culture of *R*. *rattus*, and STs of two isolates in Kandy and an isolate in Girandurukotte were ST143 and ST144, respectively (Table 1 and Fig 1).

## Discussion

This study revealed that genetically diverse *Leptospira* strains currently cause human leptospirosis in Sri Lanka. MLST showed 15 STs among 27 isolates: 6 STs have been reported in Sri Lanka, 3 STs have been found in other countries: ST30 from a human in Australia (1951), ST34 from humans in Laos (2006 and 2008) and Thailand (2000–2006) and from bandicoot rats in Thailand (2004), and ST143 from humans in Laos (2007), Thailand (2002–2004), and Russia (2014) and rats from China (1988), Indonesia (1938), and Japan (2005, 2008 and 2010), and six STs were identified for the first time in this study [20 and the MLST database (https://pubmlst.org/leptospira/)]. Various *Leptospira* genotypes (STs) were found in the Western and Southern Provinces in this study (Table 1 and Fig 1). This finding is in contrast to the previous studies based on nucleotide sequences of a single gene (16S rRNA gene) that indicated that single genotype was predominant in certain regions [13, 16]. This discrepancy may be related to their restricted sampling areas and difference in discriminatory power of MLST and 16S rRNA gene sequencing analysis.

*Leptospira interrogans* serogroup Pyrogenes ST75 has been found only in Sri Lanka since 2006 [MLST database (https://pubmlst.org/leptospira/)] and six of the seven loci are identical between ST75 and ST308 (Fig 2 and S2 Fig), indicating that new ST308 emerged from ST75. However, the way of emergence of other new STs remains unclear (Fig 2 and S2 and S3 Figs). Although they may have existed in the country for some time, the importation of livestock or unintentional introduction of rodent reservoirs via merchant ships may be the reason for the emergence of new *Leptospira* genotypes. Recent whole genome sequencing study (WGS) suggested that *L*. *interrogans* serovar Copenhageni may have spread globally via reservoir rats, *R*. *norvegicus* [28]. *L*. *interrogans* ST34 found in this study was isolated from bandicoot rats (*Bandicota indica* and *B*. *savilei*) in Thailand [29], which may have been introduced to and then spread to the indigenous bandicoot rats in Sri Lanka, but more comprehensive analysis like WGS of Sri Lankan and Thai isolates of ST34 needs to verify this assumption.

In this study, we found that *L*. *interrogans* serogroup Pyrogenes ST75 is the dominant clone in Sri Lanka (8/24). This ST strain was isolated from four mild and three severe patients (and one patient without clinical records; S1 Table). In addition, its genetically related ST308 was also isolated from severe patients, suggesting that these clonal complex strains can potentially cause severe leptospirosis in humans. These results suggest that the expansion of these clonal complex strains may be related to recent frequent outbreaks of leptospirosis. Besides ST308, novel STs, ST313 and ST314 were also isolated from severe patients during the outbreak from 2015 to 2017 in Elpitiya, Southern Province [9] (S1 Table). The relationship between *Leptospira* genotypes and their virulence has been suggested: a specific clone belonging to *L*. *interrogans* serovars Copenhageni and Autumnalis has been shown to be associated with severe pulmonary hemorrhage syndrome in Brazil and outbreaks of leptospirosis in Thailand, respectively [29, 30]. Besides bacterial virulence, the association between serum levels of cytokines and clinical outcome in patients with leptospirosis has been demonstrated [31]. Although the number of patients infected with the clonal complex strains is limited and more cases are needed, comprehensive proteomic, transcriptomic, and metabolomic analysis of blood or immune cells in mild and severe patients infected with this *L*. *interrogans* serogroup Pyrogenes clonal complex would give insights into the pathogenesis and disease severity of leptospirosis.

Although the isolates were from different provinces, *L*. *borgpetersenii* ST144 was isolated both from a human patient and a black rat, indicating that the black rat is likely to be the infection source of this strain for humans. However, there is no information on reservoir animals for other STs. As mentioned above, bandicoot rats are likely to be reservoir animals for ST34. DNA of *L*. *borgpetersenii*, *L*. *interrogans*, and *L*. *kirschneri* has recently been detected in cattle, suggesting its importance as a reservoir animal in Sri Lanka [14, 19]. In addition, antibodies against serogroup Pyrogenes were detected in buffaloes and buffalo farmers in Homagama area, Western Province [32]. Six *L*. *interrogans* serogroup Pyrogenes ST30 (2) and ST75 (4) were isolated from patients in the Base Hospital Homagama, implying buffaloes as an important reservoir in Homagama and suburbs. *L*. *interrogans* DNA and DNA of unknown *Leptospira* species have also been detected in elephant and buffalo urine samples, respectively [33, 34]. Furthermore, *Leptospira* strains belonging to serogroups Autumnalis, Hebdomadis, and Grippotyphosa without species identification were isolated from *Rattus* sp, dogs, or *Bandicota bengalensis* (*Gunomys gracilis*) [21]. In this study, the isolation rate of *Leptospira* spp. from small mammals was less than that previously described: *Leptospira* spp. were isolated from 49 of 592 (8.3%) rodents of five species about 50 years ago [21]. *Leptospira* DNA was detected from 11% rodents by real-time PCR [34]. Low carriage rate in this study may be related to environmental differences in the areas and/or season investigated. *L*. *interrogans* was linked to humid habitats, whereas *L*. *borgpetersenii* was abundant in both humid and dry habitats [35]. A higher prevalence during the dry season than rainy season has been observed in some rodent species [36]. Further investigations on cattle and other animals as well as the importance of rodents as a reservoir animal are necessary to identify reservoir animals and the transmission route(s) of leptospirosis in Sri Lanka.

In summary, genetically diverse *Leptospira* strains, both known and novel genotypes, currently cause human leptospirosis in Sri Lanka. The number of reported cases of leptospirosis with new clinical entities has increased in recent times. Three new genotypes, ST308, ST313, and ST314, were isolated from severe patients during the outbreak from 2015 to 2017 in Elpitiya, Southern Province [9]. Further studies are required to verify the relationship between the emergence of new *Leptospira* genotypes and the recent surge of leptospirosis patients and frequent outbreaks of leptospirosis in Sri Lanka.

## Supporting information

S1 Table Demographic, clinical, and bacteriological characterization of severe leptospirosis patients. (DOCX)

S1 Fig MST of 167 *L*. *borgpetersenii* strains based on the allelic profiles of MLST. Each circle represents an individual ST and circle size corresponds to the number of isolates in each ST. The length and thickness/dot of lines indicate the distance between the circles: a thicker line indicates a closer distance than a thin line, and a thin line denotes closer distance than a dotted line. The red-colored pie charts/circles and ST numbers in red represent *L*. *borgpetersenii* isolates analyzed in this study.
(PPTX)

S2 Fig MST of 681 *L*. *interrogans* strains based on the allelic profiles of MLST. Each circle represents an individual ST and circle size corresponds to the number of isolates in each ST. The length and thickness/dot of lines indicate the distance between the circles: a thicker line indicates a closer distance than a thin line, and a thin line denotes closer distance than a dotted line. The red-colored pie charts/circles and ST numbers in red represent *L*. *interrogans* isolates analyzed in this study.
(PPTX)

S3 Fig MST of 74 *L*. *kirschneri* strains based on the allelic profiles of MLST. Each circle represents an individual ST and circle size corresponds to the number of isolates in each ST. The length and thickness/dot of lines indicate the distance between the circles: a thicker line indicates a closer distance than a thin line, and a thin line denotes closer distance than a dotted line. The red-colored pie charts/circles and ST numbers in red represent *L*. *kirschneri* isolates analyzed in this study.
(PPTX)
