# Supplementary material for: Multilocus sequence typing reveals diverse known and novel genotypes of Leptospira spp. circulating in Sri Lanka
Source: PLoS Negl Trop Dis. 2020 Aug 25;14(8):e0008573. doi: 10.1371/journal.pntd.0008573 (PMC7473516; doi:10.1371/journal.pntd.0008573)
Supplement: S1 Table — (DOCX) [file pntd.0008573.s001.docx]

S1 Table. Demographic, clinical, and bacteriological characterization of severe leptospirosis patients

| Age (years) | Sex | Province | Clinical information | *Leptospira* isolate | | |
| --- | --- | --- | --- | --- | --- | --- |
|  |  |  |  | Species | Serogroup | ST |
| 12* | M | WP^†^ | AKI^†^, thrombocytopenia | *L. interrogans* | Pyrogenes | 75 |
| 45* | M | SP^†^ | AKI, pulmonary hemorrhage, thrombocytopenia | *L. interrogans* | Pyrogenes | 308^‡^ |
| 39 | M | SP | Myocarditis, shock | *L. interrogans* | Unidentified | 313^‡^ |
| 35 | F | SP | Liver failure, thrombocytopenia | *L. interrogans* | Pyrogenes | 75 |
| 20 | M | SP | AKI, thrombocytopenia | *L. interrogans* | Pyrogenes | 75 |
| 72* | M | SP | AKI, liver failure, shock, thrombocytopenia | *L. interrogans* | Autumnalis | 314^‡^ |
| 52 | M | SP | Abnormal liver function tests, thrombocytopenia | *L. interrogans* | Pyrogenes | 308^‡^ |
| 40* | M | WP | AKI, thrombocytopenia | *L. interrogans* | Autumnalis | 34 |

*The patient stayed at the intensive care unit.

^†^WP, Western Province; SP, Southern Province; AKI, acute kidney injury.

^‡^STs newly identified in this study.
