# Supplementary figures and images for: Multilocus sequence typing reveals diverse known and novel genotypes of Leptospira spp. circulating in Sri Lanka
Source: PLoS Negl Trop Dis. 2020 Aug 25;14(8):e0008573. doi: 10.1371/journal.pntd.0008573 (PMC7473516; doi:10.1371/journal.pntd.0008573)

## Slide 1
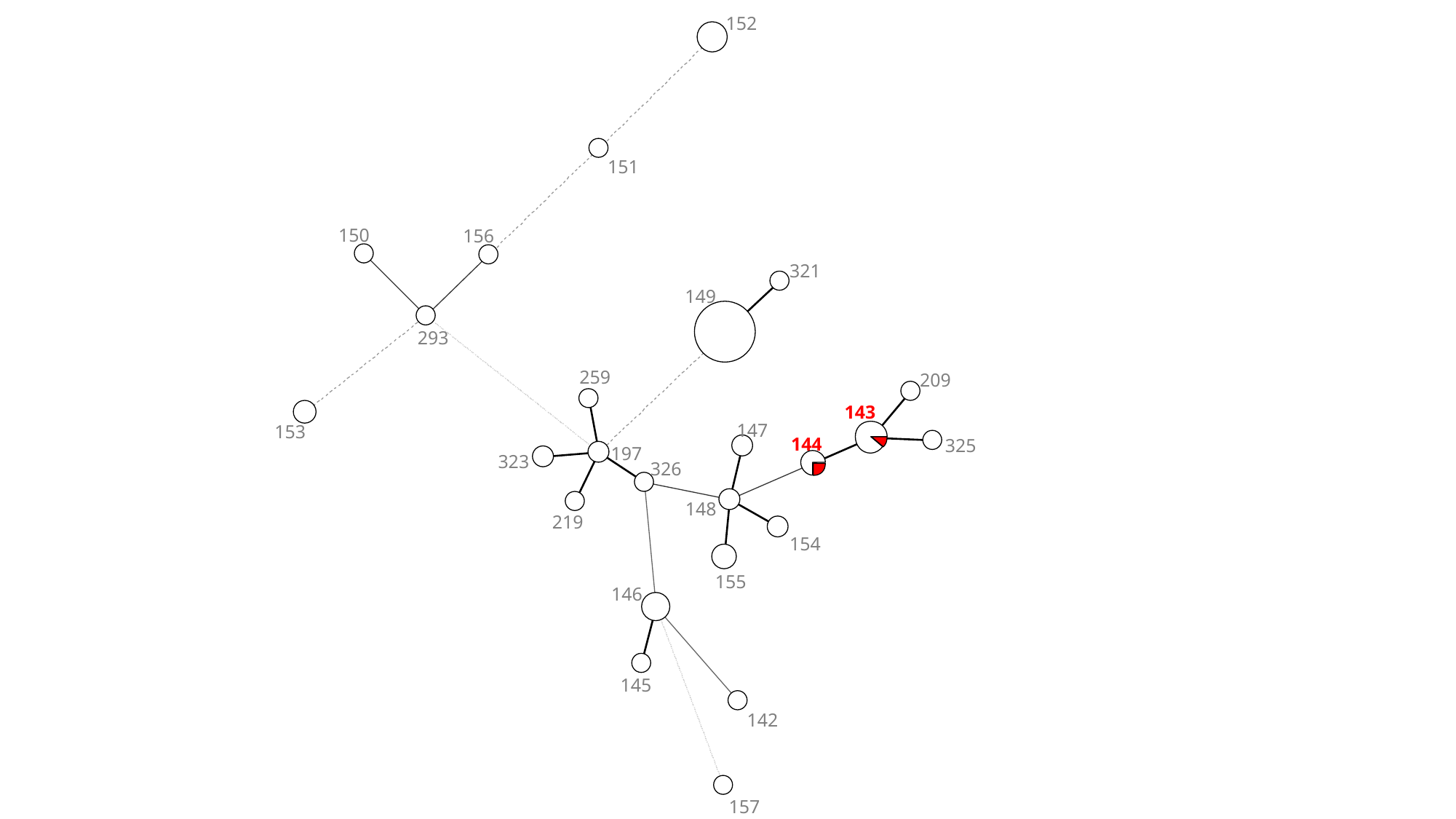

152
151
150
156
321
149
293
259
209
143
147
153
144
325
197
323
326
148
219
154
155
146
145
142
157

Supplement: S1 Fig — Each circle represents an individual ST and circle size corresponds to the number of isolates in each ST. The length and thickness/dot of lines indicate the distance between the circles: a thicker line indicates a closer distance than a thin line, and a thin line denotes closer distance than a dotted line. The red-colored pie charts/circles and ST numbers in red represent L. borgpetersenii isolates analyzed in this study. (PPTX) [file pntd.0008573.s002.pptx]

## Slide 1
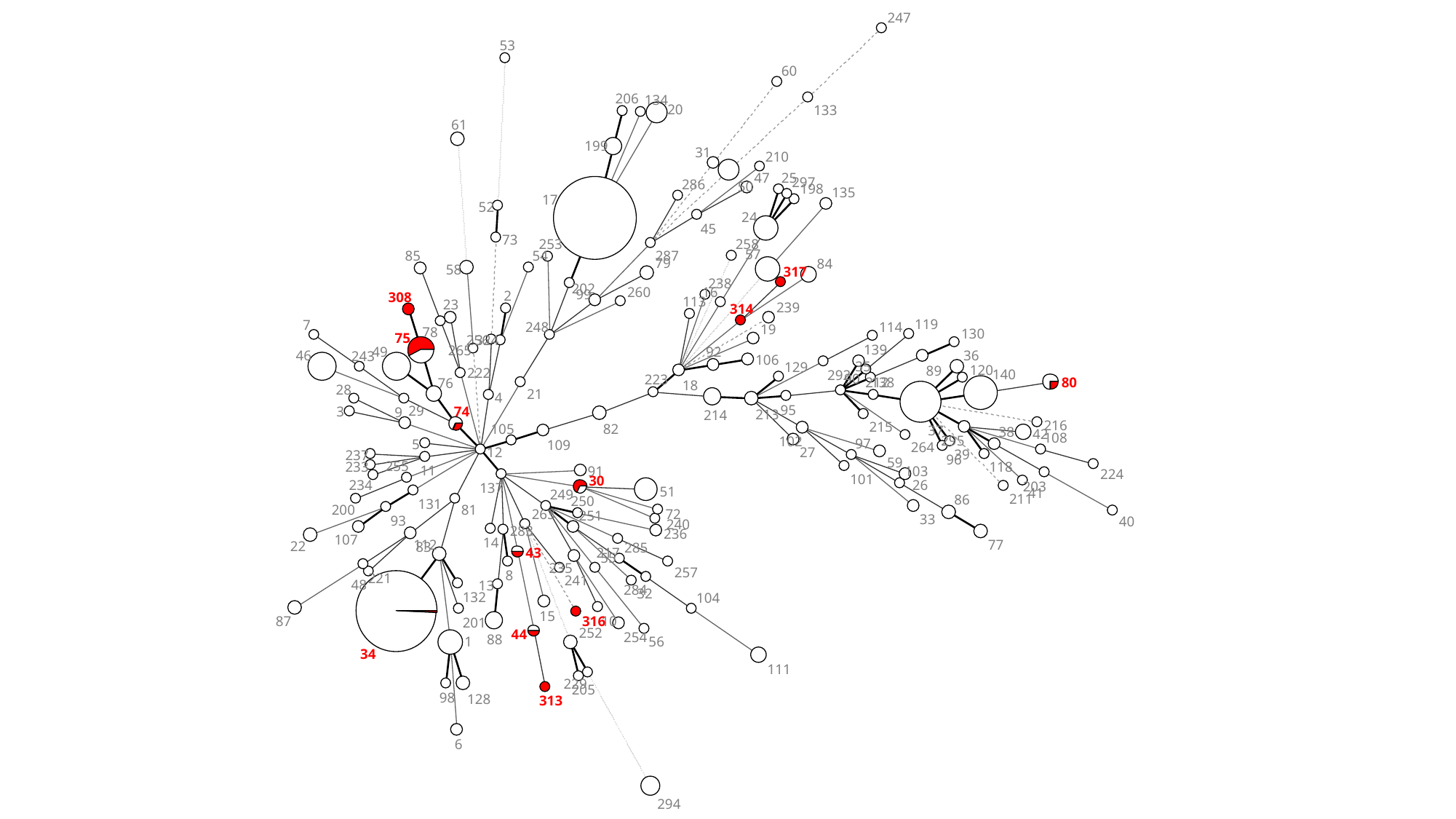

247
53
60
206
134
20
133
61
199
31
210
47
25
297
286
50
198
135
17
52
24
45
73
253
258
57
85
54
287
79
84
58
317
238
202
260
16
99
2
308
113
23
239
314
119
7
248
114
19
78
130
75
256
324
139
265
49
92
46
36
243
106
35
129
120
89
222
140
292
90
223
212
138
80
76
18
28
21
4
95
29
3
74
9
213
214
216
215
105
82
37
38
42
108
295
102
97
5
109
264
12
27
39
237
96
59
255
233
118
11
91
103
224
101
30
234
26
203
137
51
41
249
211
86
250
131
200
81
72
263
251
33
93
40
240
283
236
107
14
112
77
22
83
285
43
217
55
235
257
8
221
241
48
13
284
32
132
104
15
87
10
316
201
252
44
254
88
1
56
34
111
229
205
98
128
313
6
294

Supplement: S2 Fig — Each circle represents an individual ST and circle size corresponds to the number of isolates in each ST. The length and thickness/dot of lines indicate the distance between the circles: a thicker line indicates a closer distance than a thin line, and a thin line denotes closer distance than a dotted line. The red-colored pie charts/circles and ST numbers in red represent L. interrogans isolates analyzed in this study. (PPTX) [file pntd.0008573.s003.pptx]

## Slide 1
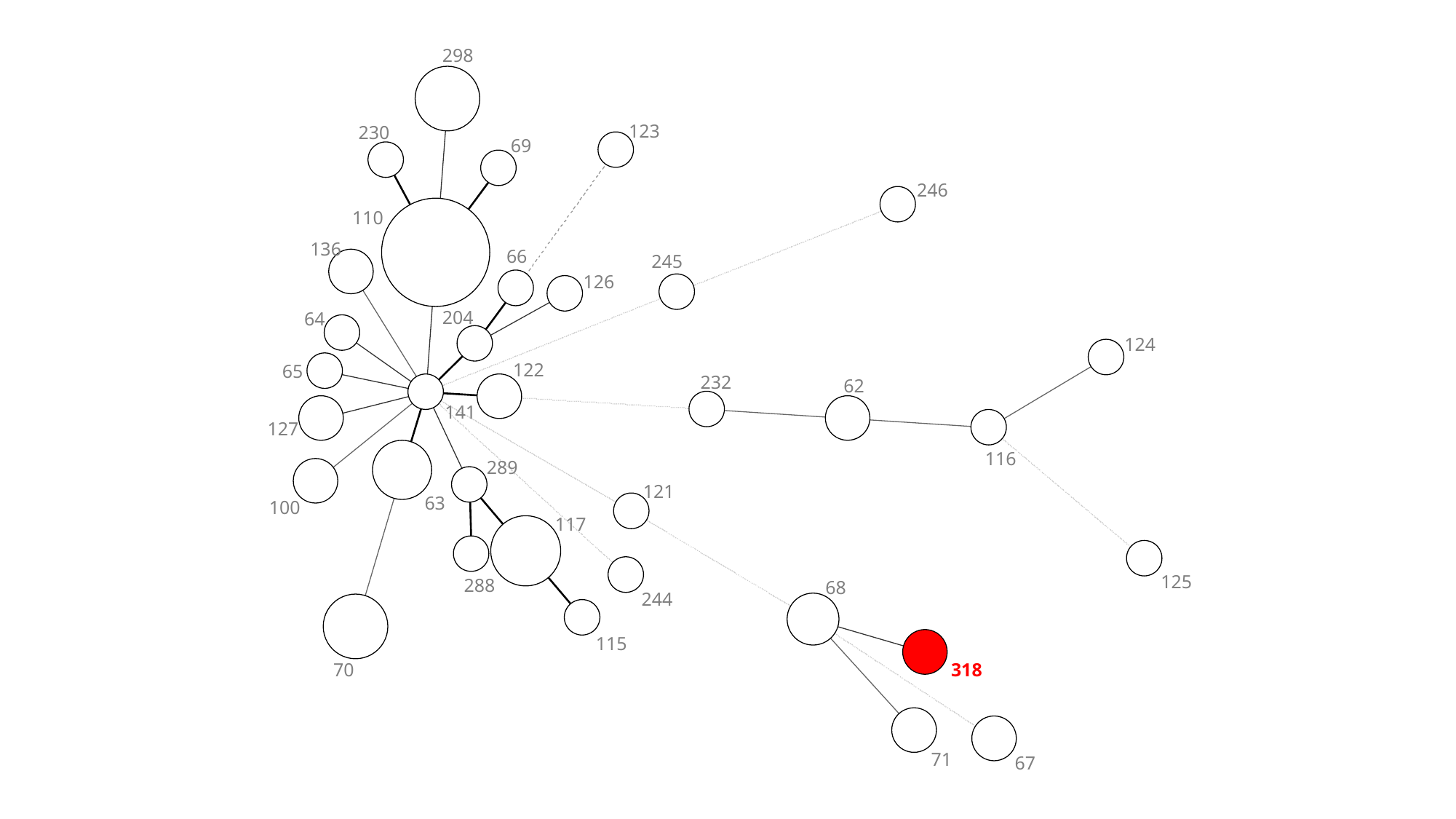

298
123
230
69
246
110
136
66
245
126
204
64
124
122
65
232
62
141
127
116
289
121
63
100
117
125
288
68
244
115
70
318
71
67

Supplement: S3 Fig — Each circle represents an individual ST and circle size corresponds to the number of isolates in each ST. The length and thickness/dot of lines indicate the distance between the circles: a thicker line indicates a closer distance than a thin line, and a thin line denotes closer distance than a dotted line. The red-colored pie charts/circles and ST numbers in red represent L. kirschneri isolates analyzed in this study. (PPTX) [file pntd.0008573.s004.pptx]
